# Cognitive control in number processing: new evidence from number compatibility effects in task-switching

**DOI:** 10.1007/s10339-022-01074-5

**Published:** 2022-02-08

**Authors:** A. Schliephake, J. Bahnmueller, K. Willmes, I. Koch, K. Moeller

**Affiliations:** 1 Leibniz-Institut Für Wissensmedien, Schleichstraße 6, 72076 Tübingen, Germany; 2 Centre for Mathematical Cognition, Loughborough University, Loughborough, UK; 3 Department of Neurology, University Hospital, RWTH Aachen University, Aachen, Germany; 4 Institute of Psychology, RWTH Aachen University, Aachen, Germany; 5 Department of Psychology, University of Tübingen, Tübingen, Germany; 6 LEAD Graduate School and Research Network, University of Tübingen, Tübingen, Germany

**Keywords:** Number processing, Cognitive control, Task-switching, Unit-decade compatibility effect, Parity congruity effect

## Abstract

A growing body of research suggests that basic numerical abilities such as number magnitude processing are influenced by cognitive control processes. So far, evidence for number processing being affected by cognitive control processes stems primarily from observed adaptations of numerical effects to stimulus set characteristics (e.g. order or ratio of specific stimulus types). Complementing previous research on adaptation to stimulus set characteristics as an index of influences of cognitive control, the present study employed a task-switching paradigm to examine how cognitive control processes influence number processing. Participants were presented with a two-digit number and had to either judge its parity or compare its magnitude to a standard depending on a preceding cue. We expected numerical congruency effects (i.e. the unit-decade compatibility effect for magnitude comparisons and the parity congruity effect for parity judgements) to be larger in switch trials, as persisting activation of the task set of the preceding trial should increase interference. In contrast to our expectations, both numerical congruity effects were reduced following task switches as compared to repetitions. This interaction of task-switching with numerical congruency effects suggests an influence of cognitive control on basic number processing in form of persisting inhibition of previously abandoned task sets, so that these exert less influence on current number processing demands.

## Introduction

Over the last decade, accumulating evidence from behavioural studies (e.g. Macizo and Herrera 2011) as well as computational modelling studies (Huber et al. 2016) suggests that processing number magnitude or place-value information (i.e. processing unit digits vs. tens digits) is adapted on a trial-by-trial basis by processes of cognitive control (e.g. Macizo and Herrera 2013). For instance, consider driving a car and suddenly you get a cue from the navigation system to turn left at the next junction. As the driver you need to switch between different tasks to resolve the situation (e.g. looking for the right junction, slowing down, putting on the indicator, etc.). Such a situation would require cognitive control to switch between tasks and execute these successfully. Similar to this example, cognitive control is required when switching between mathematical or numerical tasks, such as number magnitude comparisons or parity judgements. Notably, however, the interaction between number processing and cognitive control was predominantly evaluated in instances less complex than task switches.

Previous research considered mostly adaptations to stimulus set characteristics (e.g. the ratio of within-decade filler items; (Huber et al. 2013, 2014a, b; Macizo and Herrera 2011; Moeller et al. 2013) or item order within one single task (Macizo and Herrera 2012; Pfister et al. 2013). For instance, Macizo and Herrera (2011, 2013) evaluated alterations of the unit-decade compatibility effect (Nuerk et al. 2001) in a two-digit number comparison task. The unit-decade compatibility effect is indexed by longer reaction times and more errors for incompatible number pairs in which the larger number has the smaller unit digit (e.g. 37_52), compared to compatible number pairs for which both the comparison of tens and units lead to the same decision bias (e.g. 47_32). Macizo and Herrera (2011, 2013) observed alterations of the compatibility effect due to the proportion of within-decade filler items (e.g. 54_57). In particular, they found that higher proportions of within-decade filler items led to a more pronounced compatibility effect and agued this to reflect an increased focus on interfering unit digits due to the necessity to process the units explicitly in filler items. Hence, previous evidence suggests that number processing is affected by cognitive control processes.

Building on the empirical evidence, Huber et al. (2016) incorporated the observed effects of cognitive control as well as further evidence regarding larger multi-digit, decimal and negative numbers (e.g. Fischer 2003; Huber et al. 2014a, b; Moeller et al. 2013) in a generalized computational model framework for multi-symbol number magnitude comparison (Huber et al. 2016). This model successfully accounted for empirical evidence on influences of cognitive control processes on number processing. In particular, it proposes a weighting mechanism that assigns relative decision weights to different components of multi-symbol numbers (i.e. polarity signs as well as digits, e.g. units, tens, hundreds, etc.) in accordance with their relevance for the task at hand. For instance, in a two-digit number magnitude comparison task with only between-decade number pairs (e.g. 45_67) the weights of the tens digits are higher compared to the units digits because the tens digits are highly relevant for the overall decision, whereas the relevance of the unit digits is negligible.

So far, the model proposed by Huber et al. (2016) primarily addresses processes involved in number magnitude comparison. Although it was hypothesized that these findings should generalize to other numerical tasks and decision-making contexts, there are currently only few empirical studies providing evidence for the generalizability of the proposed influence of cognitive control on the separate processing of units, tens, hundreds, etc. to tasks other than magnitude comparison, such as parity judgements (e.g. Dehaene et al. 1993; Huber et al. 2015; Liefooghe et al. 2007). In-line with the argument on separate processing of multi-symbol numbers, we suggest that cognitive control influences on number processing (as suggested by Huber et al. 2016) are likely to generalize to tasks other than magnitude comparison. Accordingly, the adaptation of the decision relevance of processing components (i.e. units, tens, etc.) should also hold for other numerical tasks, such as parity judgements in two-digit numbers. This would indicate the generalizability of the cognitive control weighting mechanisms of the computational model by Huber et al. (2016) across different numerical tasks (i.e. number magnitude comparison and parity judgement).

The generalizability of the weighting mechanism can be evaluated considering the parity congruity effect observed in two-digit parity judgements (Dehaene et al. 1993; Huber et al. 2015). The parity congruity effect reflects a numerical congruence effect similar to the unit-decade compatibility effect, indicating faster odd/even decisions when unit and tens digits of a two-digit number are of the same parity (e.g. 24) as compared to when unit and tens digit differ in parity (e.g. 43). While the unit-decade compatibility effect is an interference effect due to the unit digit (see above), the parity congruity effect is an interference effect due to the decade digit.

Going beyond previous studies, requiring adaptations to stimulus set characteristics (see above/e.g. Macizo and Herrera 2011; Pfister et al. 2013), a more direct way of testing *how* the weighting mechanism for unit and tens digits in both number magnitude and parity judgements may be affected by cognitive control is to employ a task-switching paradigm (for recent reviews, see Kiesel et al. 2010; Koch et al. 2010, 2018; Vandierendonck, et al. 2010). In particular, the switching from one numerical task (i.e. magnitude comparison) to another (i.e. parity judgement and vice versa) goes beyond the previously employed adaptation to stimulus characteristics, as switching from one task to another requires a more active exertion of cognitive control. Task-switching paradigms involve at least two different tasks with participants performing one of these tasks on each trial. In two consecutive trials tasks are either repeated or switched. A common finding is that the processing speed of either task is reduced after a task-switch and, additionally, more errors are committed (e.g. Jersild 1927; Spector and Biederman 1976). This phenomenon is referred to as switch costs. Such switch costs were found for a variety of different tasks, different task-switching paradigms and, thus, can be considered a robust phenomenon (e.g. Kiesel et al. 2010; Vandierendonck et al. 2010).

Generally, the literature proposes at least two competing explanatory accounts through which the weighting mechanism of unit and tens digits might adapt during task-switching—persisting activation (e.g. Altmann and Gray 2008; Allport et al. 1994; Yeung and Monsell 2003) and persisting inhibition (for a review see Koch et al. 2010) of task sets in switch trials (see Monsell 2003, for a review). Both accounts are plausible explanations for the occurrence of switch costs. For instance, in the persisting activation account, a previously activated task set is argued to result in proactive interference (e.g. Allport et al. 1994; Yeung and Monsell 2003)—leading to increased reaction times in switch trials. In contrast, according to the persisting inhibition account, inhibiting the previous task is argued to include backward inhibition of response mapping rules (e.g. Regev and Meiran 2017; Schuch and Koch 2003; Schneider and Verbruggen 2008). In other words, the previous task set has to be suppressed which increases reaction times and error rates.

So far, the few previous studies employing task-switching paradigms to investigate the influence of task-switching on symbolic single-digit number processing (Schliephake et al. 2020; Wendt et al. 2013) pointed towards a stronger activation of decision irrelevant information due to interference from the previously activated task set (cf. persisting activation account). For example, in Experiment 2 of the study by Wendt et al. (2013), participants had to classify numbers according to their parity and letters according to whether they are vowels or consonants. Wendt et al. (2013; for similar results see also Pfister et al. 2013) found a reduced SNARC effect in switch trials. The SNARC effect reflects faster responses to small numbers with the left hand and large numbers with their right hand (Dehaene et al. 1993; Wood et al., 2008). Wendt and colleagues suggested that the reduced SNARC effect might be accounted for by additional control processes due to increase between-task interference in switch trials. Thus, for single-digit number processing these studies suggested that increasing demands on cognitive control reduced spatial-numerical associations irrespective of whether the additional control processes were called upon by response-related or between-task interference control.

Importantly, the task-switching paradigms in these previous studies used different stimulus types (i.e. single-digit numbers vs. letters) in the different tasks. However, for the assessment of the weighting mechanism adjusting the relevance of unit and tens digits, we suggest that employing only numerical tasks seems particularly desirable. These different numerical tasks ideally rely on the same stimulus material, while participants have to respond to different numerical features (e.g. numerical magnitude vs. parity). Employing the same numerical stimulus material allows for the control of visual information as well as the investigation of how different numerical tasks are affected in settings requiring the more active exertion of cognitive control. In addition, task switches between magnitude comparison and parity judgement always require active adjustment of the primarily decision relevant digit from the tens digit (relevant in magnitude comparison) to the unit digit (relevant in parity judgements) and vice versa through the assumed weighting mechanism as suggested by Huber et al. (2016). For instance, when switching from parity judgement to magnitude comparison or vice versa, interference from the previous trial should increase the weight of the decision irrelevant digit, because the decision weight of the irrelevant digit may be increased by the still activated task of the preceding trial.

## The present study

In the current study we employed a task-switching paradigm, in which participants were presented with a two-digit number. Depending on the colour of a respective cue, participants had to either judge whether the number was larger or smaller than 55 or whether it was odd or even. By systematically controlling repetition and switch trials (i.e. for frequency, problem size, and numerical distance between successive numbers) this study provides new insights into *how* the weighting mechanism of units and tens digits may be adjusted in situations requiring task-switching and thus the active exertion of cognitive control—in contrast to previous studies in which participants adapted to stimulus set characteristics (e.g. Herrera and Macizo 2011, 2013).

In particular, the unit-decade compatibility and the parity congruity effect should be modulated systematically by task-switching. Building on evidence from single-digit number processing pointing towards the persisting activation account on task-switching costs (e.g. Wendt et al. 2013), there should be an interfering effect of the task set activated in the previous trial on the current trial (e.g. Pfister et al. 2013). This means that the still activated task set of primary tens digit relevance for magnitude comparison in the previous trial should interfere with the primary relevance of unit digits for the current parity judgement trial and vice versa. Thus, switches from magnitude comparison to parity judgement and vice versa also require switches between the relevant digits.

Accordingly, we expected to observe larger unit-decade compatibility and parity congruity effects in switch as compared to repetition trials. Hence, the design of our study provides a possibility to gain further insights into how the weighting mechanism of two-digit number processing is affected by the exertion of cognitive control as reflected by task-switching through the hypothesized modulations of the unit-decade compatibility and parity congruity effects. Furthermore, this study might allow to gather first evidence on whether it is persisting activation that influences the weighting of tens and units in two-digit number processing.

## Method

### Participants

A priori power analyses were conducted using G*Power (Faul et al. 2009). To power for the interaction of compatibility/congruity and task-switching, analyses were run using the option ‘ANOVA: repeated measures, within-between interaction’ (with number of groups and number of measurements set to 2) to arrive at a conservative sample size estimate. Assuming a correlation of 0.7 among repeated measures, a statistical power of 0.8, and alpha of 0.05, a sample size of N = 60 is required to detect a small- to medium-sized effect of *η*_p_^2^ = 0.02 (f = 0.14). To compensate for possible attrition and to account for task permutations, data were collected from 64 participants.

The data of 57 out of 64 participants were considered for analysis (45 women, mean age = 24.7 years, SD = 4.3 years). Data of two participants were excluded for exceeding error rates of 50% in at least one block. Data of the other five participants were excluded as their overall error rates exceeded 30% (50% was guessing rate). Of the remaining 57 participants 52 reported to be right handed and the remaining five were left handed. All participants were students from different majors and reported normal or corrected to normal visual acuity. Participation was voluntary and was compensated with 6 € or course credit. The study was approved by the local ethics committee of the Leibniz-Institut für Wissensmedien, Tübingen.

### Materials

Two-digit Arabic numbers from 23 to 87 served as stimuli for the current experiment. Multiples of five, (e.g. 45), multiples of ten (e.g. 30), and ties (e.g. 33) were not included in the set. This resulted in a total of 48 stimuli, which were employed in both the magnitude comparison as well as the parity judgement task. Stimuli were presented using font Times New Roman, size 20 (resulting in a height of approximately 2.5 cm) in black against a white screen at the centre of a 21-inch computer monitor driven at a resolution of 1920 x 1200 pixels. Viewing distance was approximately 60 cm. The experiment was programmed using Experiment Builder software (SR Research, Ottawa, Canada). All stimuli are available in the online supplementary material.

Furthermore, we controlled for interference by previous stimuli caused by the rapid succession of stimuli (e.g. Neely 1977). For numerical stimuli this means considering the magnitude of the number in the previous trial, as it might interfere with processing of number magnitude in the current trial (e.g. Nuerk et al. 2005). Therefore, we balanced the frequency of all possible transitions between numbers, parity, and magnitude judgements in switch trials (i.e. repetition-switch, switch-repetition) and controlled for influences of problem size differences and numerical distance between successive numbers. Moreover, both parity judgements and magnitude comparisons comprised the same number of congruent/compatible and incongruent/incompatible number pairs. We displayed each number/stimulus twice in single-task blocks (i.e. number magnitude comparison and parity judgement, respectively) and eight times in switch blocks.

### Task and procedure

Participants had to categorize numbers as either smaller or larger than 55 (magnitude comparison task) and odd or even (parity judgement task). Responses had to be given manually by pressing either the “A” or “L” key of a QWERTZ keyboard using the left and right index fingers, respectively. Instructions focussed on both speed and accuracy.

Each participant completed one single task block of magnitude comparison and parity judgement, respectively. Afterwards, participants completed one block in the switch condition. Single-task blocks consisted of 96 trials each and the switch blocks consisted of 386 trials. As there is evidence that magnitude comparison and parity judgements are influenced by the laterality of response hands (e.g. Dehaene et al. 1993; Huber et al. 2015 for two-digit numbers), each block in the single-task and switch condition was split in two halves for which hand-to-response assignment was changed so that “smaller” and “larger” as well as “odd” and “even” responses were balanced across response hands. This means that for half of the experiment participants had to press the “A” key for “smaller” and “odd” decisions and the “L” key for “larger” and “even” decisions, before this was changed to the “L” key for “smaller” and “odd” decisions and the “A” key for “larger” and “even” decisions or vice versa. Order of hand-to-response assignments was counterbalanced across participants. At the beginning of the experiment, and after hand-to-response assignment was changed, participants had to perform three practice trials.

In the single-task block, each of the 48 stimuli was presented once per half. For instance, in magnitude trials, in the first half of the block a right-hand response indicated that the number is larger than 55 and in the second half a right key response indicated that number was smaller than 55. Block order was counterbalanced across participants.

In the switch task block, each stimulus was presented twice per half, once in a parity judgement and once in a magnitude comparison trial. Moreover, the switch block consisted of 192 repetition and 192 switch trials. In both magnitude comparison and parity judgement trials hand to response assignment was reversed in the second half of the switch task block to control for potential spatial-numerical associations of smaller/larger numbers and left/right response hand. Block order was counterbalanced across participants. Parity judgement and magnitude comparison trials were presented in pseudorandom order. Switch and repetition trials had an identical frequency of magnitude comparison and parity judgement trials and repetitions and switches between magnitude comparison and parity judgement trials were counterbalanced. Each trial type (i.e. repetition and switches) occurred no more than four times in a row. Four pseudorandom trial sequences were generated prior to the experimental session.

**Fig. 1:**
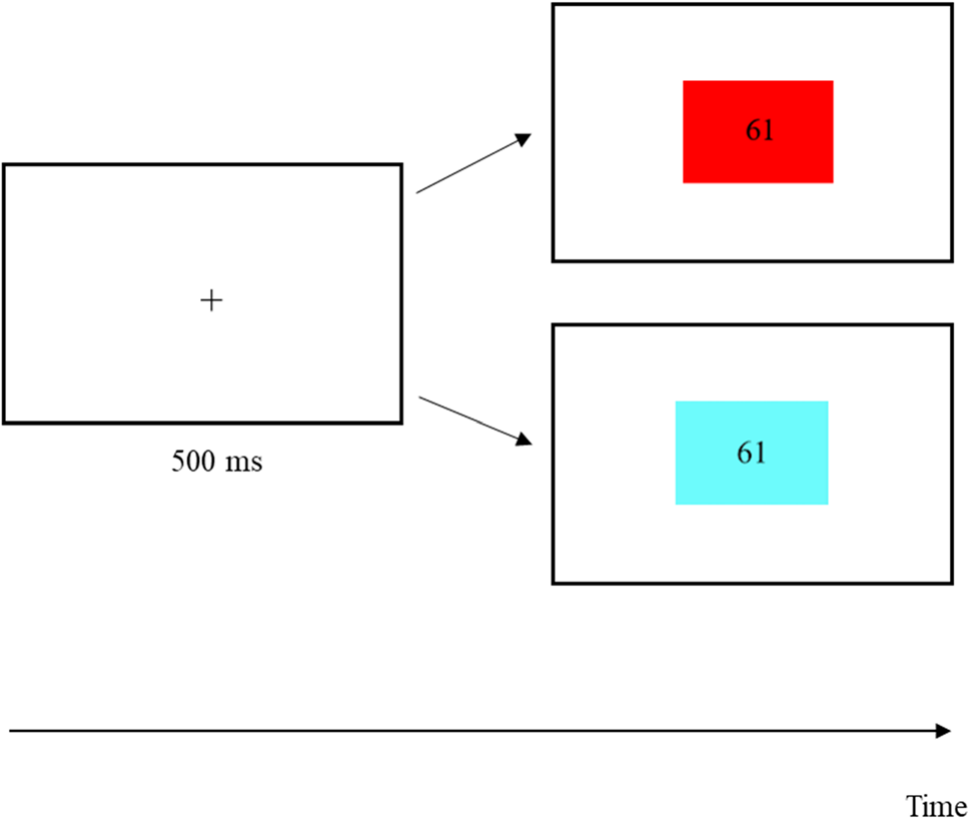
Exemplary depiction of a switch trial. *Note.* After the fixation cross was displayed for 500 ms the participant either had to judge the parity (indicated by a turquoise cue) or compare the magnitude (indicated by a red cue) of the displayed number to the standard 55. Please note that hand to response assignment (i.e. whether “odd/even” or “smaller/larger” decision had to be indicated by pressing the “left/right” response button) was reversed at the middle of each block

Each trial started with a fixation cross that was displayed for 500 ms, followed by stimulus presentation and ending with the response or after a maximum response interval of 4000 ms (see Fig. 1 for a depiction of a trial). Both fixation cross and stimulus were presented in the centre of the screen. In the switch block, a red or cyan square was displayed in the background of each number with square and number appearing at the same time. A red square indicated a magnitude comparison to be performed on the number presented, whereas a cyan square indicated a parity judgement to be done on the number presented. Intervals from stimulus onset to response were considered as RT. A response was followed by the start of the next trial after a response-stimulus interval of 500 ms. Each session lasted approximately 40 min. At the beginning of the experiment, and after hand-to-response assignment was changed, participants had to perform three practice trials.

### Design

We investigated the effects unit-decade compatibility (compatible vs. incompatible) and parity congruity (congruent vs. incongruent) and how they are modulated by task-switching (repetition vs. switch). The parity congruity effect was only assessed for parity judgement trials and the unit-decade compatibility effect only for magnitude comparison trials, that is, we have two separate 2 (compatibility/congruity) × 2 (task-switch) designs, one for each effect. Errors were infrequent and below 10% per participant. The general pattern in the error rates was similar but less differentiated than the pattern of RT effects. Therefore, the error rates will not be reported.

## Results

### Analysis

For the analyses, we focussed on items from 23 to 49 and 61 to 87 (N = 40) as those can be classified as either unit-decade compatible or incompatible with respect to the comparison standard 55. Additionally, we excluded items following items from 51 to 59 because we were interested in effects of repetition vs. switch.^1^ Prior to analyses, error trials, practice trials, non-responses (due to time out), and the first trial of each switch block half (as these are neither repetition nor switch trials) were eliminated. Fixed cut-offs of RT < 200 ms and RT > 2500 were applied to eliminate premature or extraordinary long responses. RT outliers of ± 3 SD around individuals’ mean RT were also eliminated from the data set. In total, data pre-processing resulted in a loss of approximately 12.6% of the data. To approximate normal distribution RT data were log transformed prior to the analysis. For reasons of comprehensibility, we report raw RT when describing significant results. Data preparation and analyses were done in R (R Core Team 2020) with the afex (Singmann et al. 2019) and data table (Dowle and Srinivasan 2020) packages.

We evaluated expected effects of unit-decade compatibility and parity congruity and the modulation of the numerical effects through task-switching by conducting one 2 × 2 within-participant ANOVA per numerical effect. These included the independent variables task-switching (repetition vs. switch) for both unit-decade compatibility (compatible, i.e. 24 vs. incompatible, i.e. 28) and parity congruity (congruent 46 vs. incongruent 27), respectively. When the assumption of sphericity was not met, we applied the Greenhouse–Geisser correction. In this case, the Greenhouse–Geisser coefficient (GG) for adjusting the respective degrees of freedom is reported. Significant ANOVA interactions were followed-up by univariate ANOVAs to evaluate simple effects. For the interested reader additional analysis of the single task blocks (reporting on the unit-decade and parity congruity effect, respectively, without task-switching requirements) as well as analysis on so-called mixing costs contrasting the single task blocks with the repetition condition and a brief discussion of these are provided in the Appendix.

### Modulation of the unit-decade compatibility effect

#### Switch costs

Task-switching (repetition vs. switch) had a significant main effect on RT [*F*(1,56) = 211.66, *p* < 0.001; *η*_p_^2^ = 0.79] with significantly shorter RT in the repetition trials (*M* = 817 ms, *SD* = 159 ms) than in switch trials (*M* = 1042 ms, *SD* = 223 ms). 55/57 (96.5%) of the participants showed longer RT in switch trials. There was a significant main effect of unit-decade compatibility on RT [*F*(1, 56) = 9.72, *p* < 0.001; *η*_p_^2^ = 0.15] with 25 ms shorter RT for compatible trials (*M* = 917, *SD* = 238) as compared to incompatible trials (*M* = 942, *SD* = 220). Furthermore, the respective compatibility effect was positive in 38/59 participants (63.2%).

**Fig. 2:**
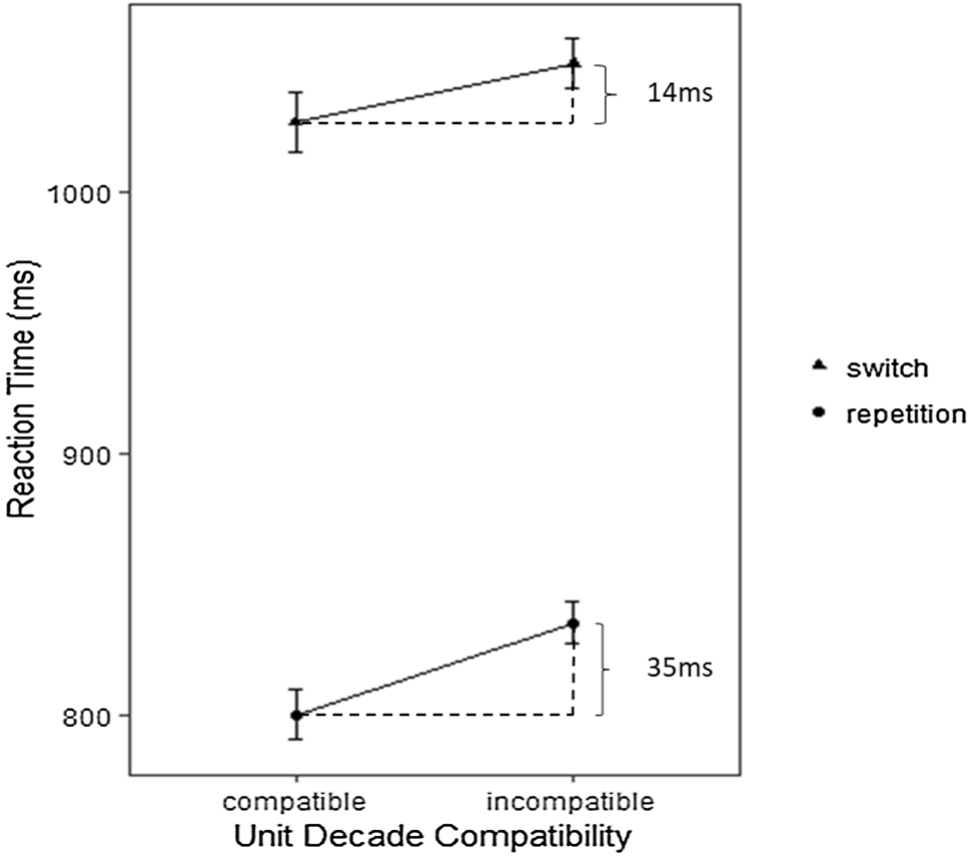
Illustration of the interaction of the unit-decade compatibility (for magnitude comparison) depicting a larger compatibility effect (35 ms) for repetition as compared to switch trials (14 ms). Error bars indicate 1 standard errors of the mean (SEM)

The interaction between task-switching and unit-decade compatibility indicated a non-significant trend [*F*(1, 56) = 5.74, *p* = .09; *η*_p_^2^ =.05]. The unit-decade compatibility effect tended to be larger in repetition trials (*M* = 35 ms, *SD* = 97 ms) as compared to switch trials (*M* = 14 ms, *SD* = 106 ms). Analysis of simple effects indicated that the unit-decade compatibility effect was significant in repetition [*F*(1, 56) = 11.25 *p* < .001; *η*_p_^2^ =.17], but not in switch trials [*F*(1,56) = 1.58, *p* = .21;*η*_p_^2^ =.02]. Unit-decade compatibility effects were positive in 39/57 participants (68.4%) in repetition trials and 30/57 participants (52.6%) in switch trials, respectively (Fig. 2, Table 1).

**Table 1:** Mean RTs (ms) and mean error rates (ER, %) with standard deviations (ms in parentheses) and the respective compatibility effects depending on task type and unit-decade compatibility

	Single-task	Task repetition	Task-switch
RT (ms) Compatible	596 (124)	800 (169)	1035 (240)
RT (ms) Incompatible	597 (121)	835 (164)	1049 (218)
ER (%) Compatible	1.2 (3.1)	7.5 (14.0)	9.7 (16.0)
ER (%) Incompatible	2.6 (3.9)	8.8 (14.0)	10.2 (15.3)
*Compatibility effects (incompatible- compatible)*			
RT (ms)	− 1	35	14
ER (%)	1.4	1.3	0.5

### Modulation of the parity congruity effect

#### Switch cost

Task-switching (repetition vs. switch) had a significant main effect on RT [*F*(1,56) = 223.02, *p* < 0.001; *η*_p_^2^ = 0.80] with significantly shorter RT in repetition trials (*M* = 845 ms, *SD* = 182 ms) as compared to switch trials (*M* = 1106 ms, *SD* = 226 ms) with 56/57 (98.2%) of the participants showing longer RT in switch trials. The main effect of parity congruity was also significant [*F*(1, 56) = 5.55, *p* = 0.02; *η*_p_^2^ = 0.09], indicating that mean RT was shorter for congruent trials (*M* = 967, *SD* = 242) as compared to incongruent trials (*M* = 984, *SD* = 244). The respective congruity effect was positive in 32/57 participants (56.1%).

**Fig. 3:**
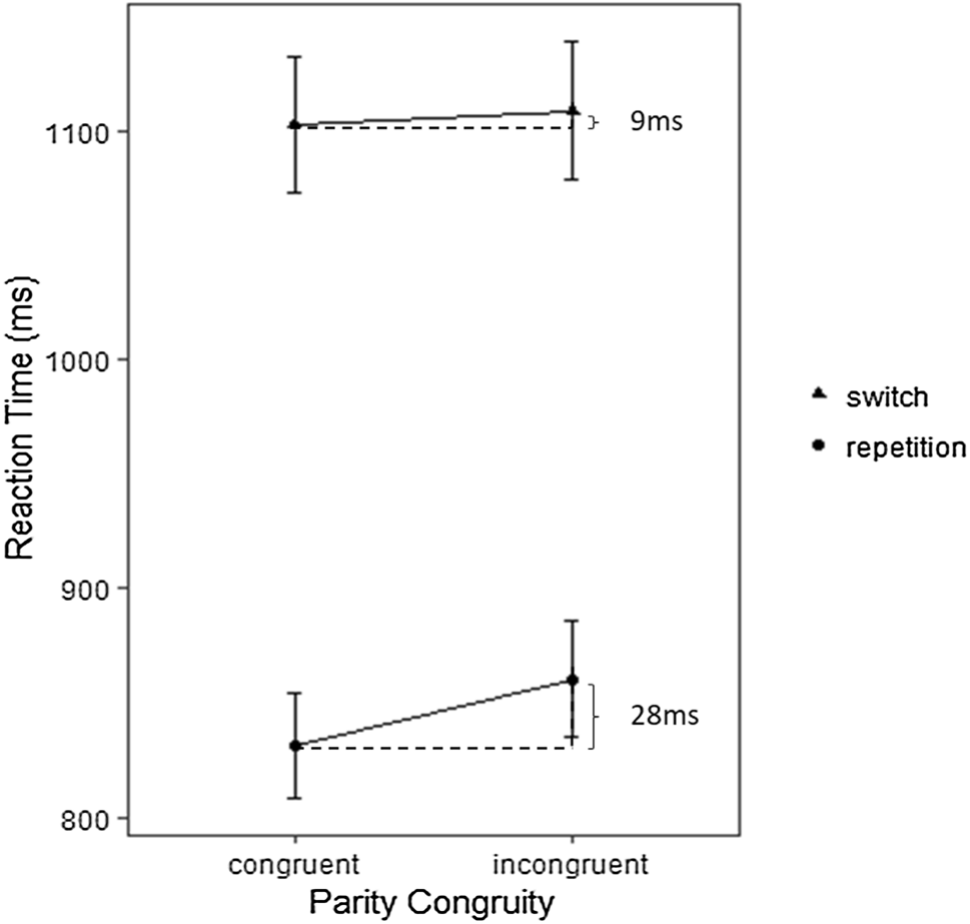
Illustration of the interaction of parity congruity (for parity judgements) depicting a larger parity congruity effect (28 ms) in repetition as compared to switch trials (9 ms). Error bars indicate 1 standard errors of the mean (SEM)

The interaction between task-switching and parity congruity was significant [*F*(1, 56) = 5.95, *p* = .018; *η*_p_^2^ =.10], indicating that the parity congruity effect was significantly larger in repetition trials (*M* = 28 ms, *SD* = 64 ms) compared to switch trials (*M* = 9 ms, *SD* = 106 ms). Analysis of simple effects indicated that the parity congruity effect was significant in repetition trials [*F*(1, 56) = 16.17, *p* < .001; *η*_p_^2^ =.22], but not in switch trials [*F*(1,56) = 0.08, *p* = .78; *η*_p_^2^ =.001]. Parity congruity effects were positive in 41/57 participants (71.9%) in repetition trials and 30/57 participants (52.6%) in switch trials, respectively (Fig. 3, Table 2).

**Table 2:** Mean RTs (ms) and mean error rates (ER%) with standard deviations (ms in parentheses) as well as the respective parity congruity effects depending on task type and parity congruity

	Single-task	Task repetition	Task-switch
RT (ms) Congruent	612 (137)	831 (172)	1103 (226)
RT (ms) Incongruent	650 (144)	860 (191)	1109 (228)
ER (%) Congruent	5.4 (10.5)	6.6 (10.2)	9.9 (12.8)
ER (%) Incongruent	6.0 (9.5)	8.0 (12.7)	11.5 (12.1)
*Parity congruity effects (incongruent–congruent*)			
RT (ms)	38	29	6
ER (%)	0.6	1.4	1.6

## Discussion

In this study, we aimed at examining *how* basic numerical processing of two-digit numbers is affected in task-switching situations, requiring the exertion of cognitive control. To do so, we used a cued task-switching paradigm in which two-digit numbers had to be classified as being smaller or larger than 55 or as being odd or even. Thereby, we investigated *how* number processing is influenced by cognitive control in two different kinds of numerical information: number magnitude (indexed by changes in the unit-decade compatibility effect, e.g. Nuerk et al. 2001) and parity (indexed by alterations of the parity congruity effect, e.g. Dehaene et al. 1993; Huber et al. 2015). We expected, based on evidence from single-digit number processing (e.g. Wendt et al. 2013; Schliephake et al. 2020) and the persisting activation account for task-switching costs (e.g. Koch et al. 2010) that switching from parity judgements to magnitude comparisons or vice versa should amplify both the unit-decade compatibility effect and the parity congruity effect, because the task set of the previous task might still be activated. Accordingly, the decision weight of the respective decision irrelevant interfering digit should be increased. In-line with our expectations, we observed a modulation of the numerical effects by task-switching. However, in contrast to our hypotheses, the parity congruity effect was significantly larger in repetition as compared to switch trials and a similar trend was observed for the unit-decade compatibility effect.

Overall, the present study provides new evidence that number processing is affected by cognitive control processes—rather than being entirely automatic—because both the processing of number parity processing as well as tendentially the processing of number magnitude processing was modulated by task-switching. In particular, the parity congruity effect was significantly larger in repetition as compared to switch trials with a similar tendency for the unit-decade compatibility effect, which was significant only in repetition but not in switch trials. Hence, the overall results pattern suggested that interference by the respective decision irrelevant digits (i.e. unit digit in magnitude comparison vs. tens digits in parity judgements) was less pronounced in switch as compared to repetition trials.

Regarding the unit-decade compatibility effect, the findings of a recent study by Petruo et al. (2019) might explain why in the present study the influence of task-switching on the parity congruity effect was significant but was less robust for the unit-decade compatibility effect. Petruo et al. (2019) showed that task-switching was more demanding during parity judgements as compared to magnitude comparisons. The authors argued that parity judgements exacerbate processes of updating and reconfiguring task sets during task-switching. Considering this in the context of our findings, it seems plausible that the higher demands during processing parity information made it more susceptible to influences of task-switching. Furthermore, Fitousi and Algom (2019) observed that participants were able to ignore the unit digit of a two-digit number but were unable to do so for the tens digit. This adds to the argument of higher processing demands and interference due to the decision irrelevant digit during parity processing, which necessarily requires processing the unit digit. Taken together, these findings might explain why we found a significant influence of task-switching on the parity congruity effect, but not on the unit-decade compatibility effect. As such, one might speculate that higher processing demands during parity processing in switch trials required stronger exertion of cognitive control, which in turn increased its effect on the parity congruity effect.

Furthermore, our findings are in contrast with what we expected based on experiments investigating single-digit number processing in task-switching settings and the persisting activation account. This new evidence points towards a decreased weight of the respective task-irrelevant digit in switch trials, indicating that irrelevant information may be inhibited rather than activated in situations requiring the exertion of cognitive control (i.e. task-switching). In other words, the present results rather support the account on persisting inhibition account of the previously activated task set. Thus, in the context of ongoing discussions contrasting persisting activation vs. persisting inhibition accounts as possible origin of switch costs (see Koch et al. 2010), our findings seem to corroborate the persisting inhibition account assuming backward inhibition of previous response mapping rules (e.g. Brown et al. 2007; Goschke 2000; Mayr and Keele 2000; Philipp and Koch 2006; Sdoia and Ferlazzo 2008).

Generally, inhibition-based accounts of switch costs do not assume spill over of task set activation from the previous task, but claim that the task set of the previous task is actively inhibited to increase performance on the actual task (e.g. Koch et al. 2010). Evidence in favour of inhibitory accounts on switch costs comes from experiments evaluating congruence effects across task switches and found that switch costs and reaction times are lower in congruent than in incongruent trials. In such experiments bivalent stimuli were employed (i.e. congruent stimuli that required the same response in both tasks vs. incongruent stimuli which required different responses depending on the task). For instance, considering the switch between shape classifications (circle vs. square) and colour discriminations (red vs. blue)—in congruent trials both the colour blue and the shape square were mapped to the same response key, as opposed to incongruent trials in which shape and colour were mapped to different response keys. As such, a blue square would be a congruent stimulus, whereas a red square would be an incongruent stimulus. Overall, in these paradigms it was observed, similar to numerical congruence effects, that reaction times are higher for incongruent stimuli than for congruent stimuli (e.g. Meiran and Kessler 2008), indicating that incongruent trials increase task- and response conflict (e.g. Koch and Allport 2006; Meiran and Kessler 2008). However, this evidence could not resolve the debate about which task-switching account might explain the occurrence of switch costs, as both persisting activation and persisting inhibition of the previous task set could equally well explain the increased reaction time in incongruent trials. In this regard, the present study evaluating influences of task-switching on numerical congruence effects might add another facet to the debate, as the (tendentially) reduced numerical congruence effects seem to reflect inhibition of the previous trial. Thus, from a number processing perspective it seems plausible, that interfering information of the previous task set (i.e. the in the current task irrelevant but in the previous task relevant digit) is actively inhibited rather than still activated.

In terms of the underlying weighting mechanism, it seems that the decision weight of the irrelevant digit is decreased systematically in switch trials. This is reflected by decreased parity congruity and no longer significant unit-decade compatibility effects in switch as compared to repetition trials—indicating that the weight of the decision irrelevant digit is reduced in switch trials. For instance, when in a previous magnitude comparison trial the tens digit was primarily decision relevant and in the consecutive parity judgement the unit digit is relevant—the weight of the tens digit seems to be specifically decreased, which is indicated by a weaker parity congruity effect.

Further evidence allowing to differentiate how the weighting mechanism adapts in situations requiring the active exertion of cognitive control might be informed by theoretical accounts on switch costs (e.g. persisting activation vs. persisting inhibition account). In particular, evidence from participants’ eye-fixation behaviour might provide further insights into which digits are focussed more frequently during task-switching (see Mock et al. 2016, for a review on eye-tracking in numerical cognition research). For instance, according to the results of Huber et al. (2014a, b), who observed more fixations on the, respectively, more relevant digit in magnitude comparisons, one would expect more fixations on the decision relevant digit in switch trials as compared to repetition trials resulting in less interference due to the irrelevant digit and, thus, smaller congruity effects in switch trials.

Moreover, there is evidence from neuropsychological studies substantiating the idea that in situations of response conflict the information needed to solve the task becomes more relevant by means of specific inhibition of irrelevant information (e.g. Egner and Hirsch 2005; Meiran et al. 2011). For instance, Egner and Hirsch (2005) observed shorter reaction times and increased neural activation in relevant brain areas for incongruent trials preceded by another incongruent trial and interpreted this as evidence for the amplification of relevant information, reflecting successful conflict resolution through cognitive control (e.g. Botvinick et al. 2001; Kerns 2004). Thus, also in situations of response conflict, the adaptation of the weighting mechanism reflecting the processing weight of the respective digits seems to align more closely with previous evidence pointing towards the persisting inhibition of distracting information.

Adding to this, the present results might help extending the existing computational model for magnitude comparisons by Huber et al. (2016) to parity judgements. In particular, this would require specifying the cognitive control processes of the model to not only consider adaptations to stimulus set characteristics, but also task switches that change the relevance of the respective digits for the overall decision. An additional task-switch node needs to adjust the relevance weighting of units and tens depending on the task at hand (i.e. decrease the weight of unit digits in magnitude comparisons versus decreasing the weight of tens digits in parity comparison). Conceptually, empirical but also computational follow-up studies have the potential to provide more information on the exact working mechanisms of cognitive control in number processing.

In conclusion, our findings provide first insights into how the weighting of decision relevant and irrelevant digits of two-digit numbers might be affected by cognitive control. This generalizes previous effects exclusively observed for number magnitude processing to the processing of parity information. In particular, the present results indicate that in situations that require more active exertion of cognitive control (e.g. manipulating the decision relevant digit of a multi-digit number in a numerical task-switching paradigm) yield respective adjustments of the decision weights of units and tens through seemingly persisting inhibition of decision irrelevant digits.

## Data Availability

The datasets generated during and/or analysed during the current study are not publicly available, as the sharing of data publicly was not covered by the informed consent. The data is available from the corresponding author on reasonable request.

## Appendix

As common in task-switching designs, participants completed blocks with the single tasks before the task-switch condition which we will report in the following. For the interested reader, some remarks may be allowed regarding inferences drawn from these additional data.

In addition to switch costs, considering the data from single task blocks and repetition trials from the switch block also mixing costs (Los 1996) can be evaluated. Mixing cost represent differences in response time and accuracy between a single-task condition (in which only one task has to be completed by participants) and the repetition condition. On a conceptual level, mixing costs are argued to allow inferences about the involvement of working memory processes in repetition trials, as two task sets need to be kept in mind (e.g. Koch 2005). This is the case as single-task block trials do not require the shifting between task sets at all, whereas repetition trials require at least the anticipation of a potential task set shift for the next trial (Miyake et al. 2000). Put differently, comparing single-task trials and repetition trials allows to specifically evaluate effects of task set updating and maintenance. As such, mixing costs were advocated as another measure of cognitive control processes (Marí-Beffa and Kirkham 2014).

However, for the assessment of numerical congruency effects there are certain objections against a comparison of the single task and the repetition condition, as for instance the unit-decade compatibility effect seems to be less stable in situations requiring little cognitive effort—as it is the case in the single task condition—and depend on the stimulus set composition (e.g. Nuerk and Willmes 2005; Moeller et al. 2009). In particular, previous studies indicated that the unit-decade compatibility effect was modulated by unit distance (i.e. the difference between the unit digits of the to-be-compared numbers) with smaller or absent compatibility effects for smaller unit digit distances (e.g. Moeller et al. 2009, Nuerk et al. 2001, see also Zhang and Wang 2005). In the following we present mixing costs analysis for magnitude comparisons and parity judgements and a brief discussion of the results.

### Supplementary unit-decade compatibility effect analysis

#### Mixing costs

Task mixing (single-task vs. repetition) had a significant main effect [*F*(1, 56) = 207.39, *p* < 0.0001; *η*_p_^2^ = 0.79] with significantly shorter RT in the single-task block (*M* = 596 ms, *SD* = 122 ms) than in the repetition trials (*M* = 817 ms, *SD* = 167 ms). 56/57 (98.2%) of participants showed longer RT in repetition trials. Furthermore, there was also a significant main effect of unit-decade compatibility on RT [*F*(1, 56) = 6.43, *p* = 0.01; *η*_p_^2^ = 0.10] indicating shorter RT for compatible (*M* = 698 ms, *SD* = 180 ms) as compared to incompatible number pairs (*M* = 715 ms, *SD* = 187 ms). The respective compatibility effect was positive in 38/57 participants (66.7%).

The interaction between task mixing and unit-decade compatibility was also significant [*F*(1, 56) = 10.64, *p* < 0.01; *η*_p_^2^ = 0.16]. The unit-decade compatibility effect was significantly smaller in single-task trials (*M* = -1 ms, *SD* = 55 ms) as compared to repetition trials (*M* = 35 ms, *SD* = 97 ms). Analysis of simple effects indicated that the unit-decade compatibility effect was not significant in the single-task block [*F*(1,56) = 0.001, *p* = 0.78 *η*_p_^2^ = 0.001, but significant in repetition trials [*F*(1, 56) = 11.25, *p* < 0.001; *η*_p_^2^ = 0.17]; see above]. Unit-decade compatibility effects were positive in 32/57 participants (56.1%) in the single-task block and 39/57 participants (68.4%) in repetition trials.

### Supplementary parity congruity effect analysis

#### Mixing costs

The ANOVA indicated a significant main effect for task mixing [*F*(1, 56) = 159.72, *p* < .001; *η*_p_^2^ = .74], reflecting significantly shorter RT in the single-task block (*M* = 631 ms, *SD* = 141 ms) as compared to repetition trials (*p* < .001, *M* = 845 ms, *SD* = 182 ms). 54/57 (94.7%) participants showed longer reaction times in repetition trials. Moreover, there was a significant main effect of parity congruity on RT [*F*(1, 56) = 49.68, *p* = .01; *η*_p_^2^ = .47]: mean RT was shorter for congruent number pairs (*M* = 722 ms, *SD* = 190 ms) as compared to incongruent number pairs (*M* = 755 ms, *SD* = 199 ms). The respective congruity effect was positive in 47/57 participants (82.5%).

The interaction between task mixing and parity congruity was not significant [*F*(1, 56) = 2.40, *p* < .13 ; *η*_p_^2^ =.04] indicating no differences in parity congruity effects between single-task block trials (*M* = 38 ms, *SD* = 54 ms) and repetition trials (*M* = 29 ms, *SD* = 64 ms). Simple effects indicated that the parity congruity effect was significant in single-task block trials [*F*(1,56) = 40.38, *p* < .001, *η*_p_^2^ =.42] and in repetition trials [*F*(1, 56) = 16.17, *p* < .001; *η*_p_^2^ =.22]. Parity congruity effects were positive in 46/57 participants (80.7%) in the single-task block and 41/57 participants (71.9%) in repetition trials.

## Summary and conclusion

Overall, the analysis of mixing costs, despite the significant difference of unit-decade compatibility effects in single and repetition trials, do not seem to add any further evidence for our claim on effects of cognitive control on number processing. As discussed above, the unit-decade compatibility effect was absent in the single task condition, which is probably caused by the low unit digit distance of 4 (the standard was 55, e.g. 55_79). As small unit digit distances were observed to lead to less pronounced unit-decade compatibility effects (e.g. Nuerk et al. 2001; Moeller et al. 2009) this seems to account for the non-significant effect in the single-trial condition. However, as we were interested in modulations of the unit-decade compatibility effect by task-switching, the absence of the unit-decade compatibility effect in single trials does not hamper the interpretability of our results. Consequently, for future research we encourage testing whether there is a significant modulation of the unit-decade compatibility effect when a different comparison standard with a larger unit distance is employed (e.g. a standard of 53, cf. Moeller et al. 2009) (Table 2).

## Footnotes

1 Please note that results did not differ substantially when stimuli that follow stimuli between 51 and 59 were included in the analyses.

## Code availability

The R code written for the analysis of the current study is available from the corresponding author on reasonable request.
